# 

**Published:** 1888-08

**Authors:** 


Volume LVII.] AUGUST, 1888. [Number 2.

### The CHICAGO MEDICAL JOURNAL AND EXAMINER

EDITOR:

S. J. JONES, M.D., LL.D

Rand, McNally & Company, Printers.

1888.

Entered at the Post Office at Chicago, for transmission through the mails as second-class matter.

